# Supplementary figures and images for: How does individualised physiotherapy work for people with low back pain? A Bayesian Network analysis using randomised controlled trial data
Source: PLoS One. 2021 Oct 11;16(10):e0258515. doi: 10.1371/journal.pone.0258515 (PMC8504753; doi:10.1371/journal.pone.0258515)

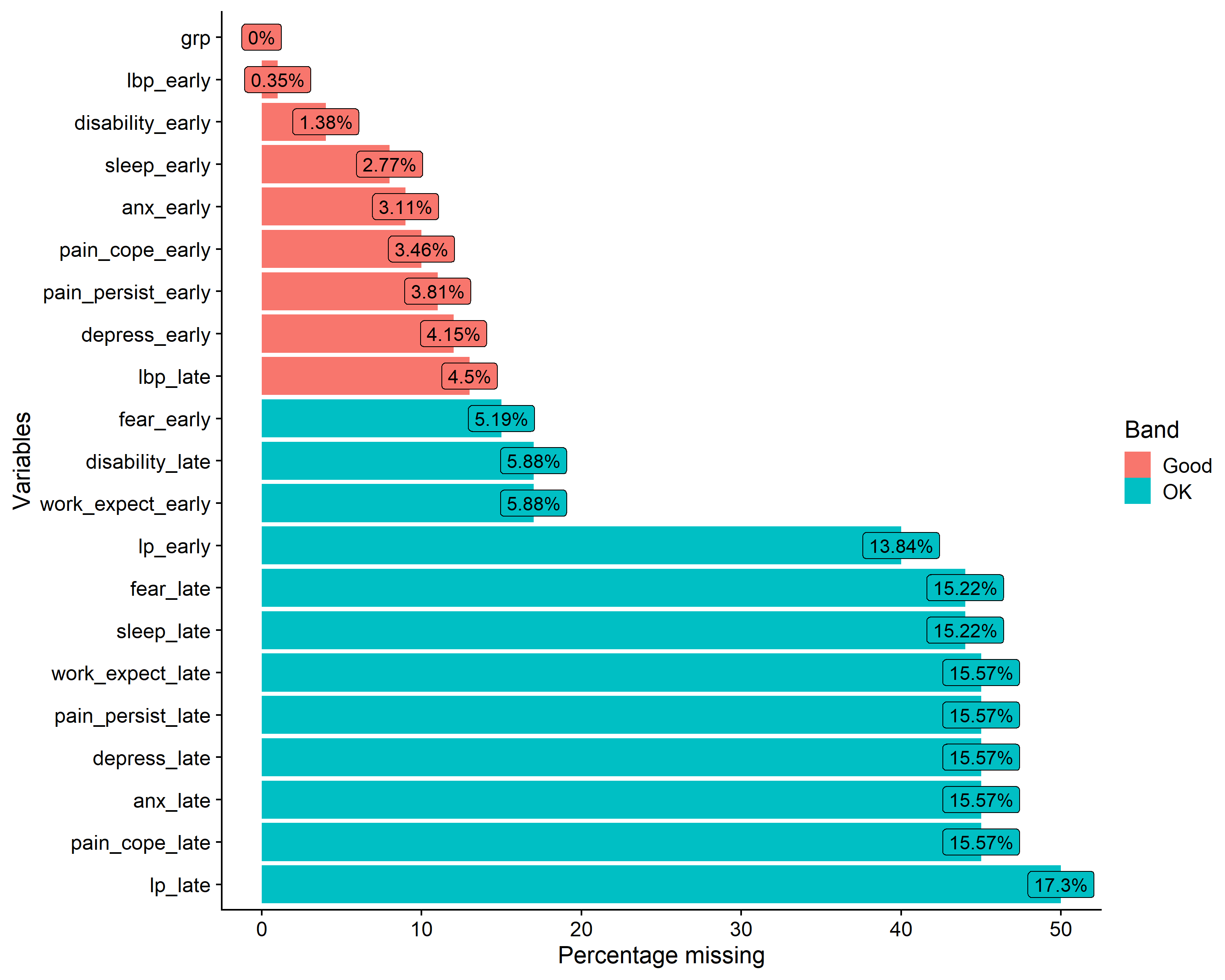

Supplement: S1 Fig — Abbreviation: Suffix with “_early”–change score between baseline and week-10 follow-up; “_late”–change value between week-10 and week-52;Anx—anxiety; grp–group; lbp–low back pain; lp–leg pain. (TIFF) [file pone.0258515.s001.tiff]

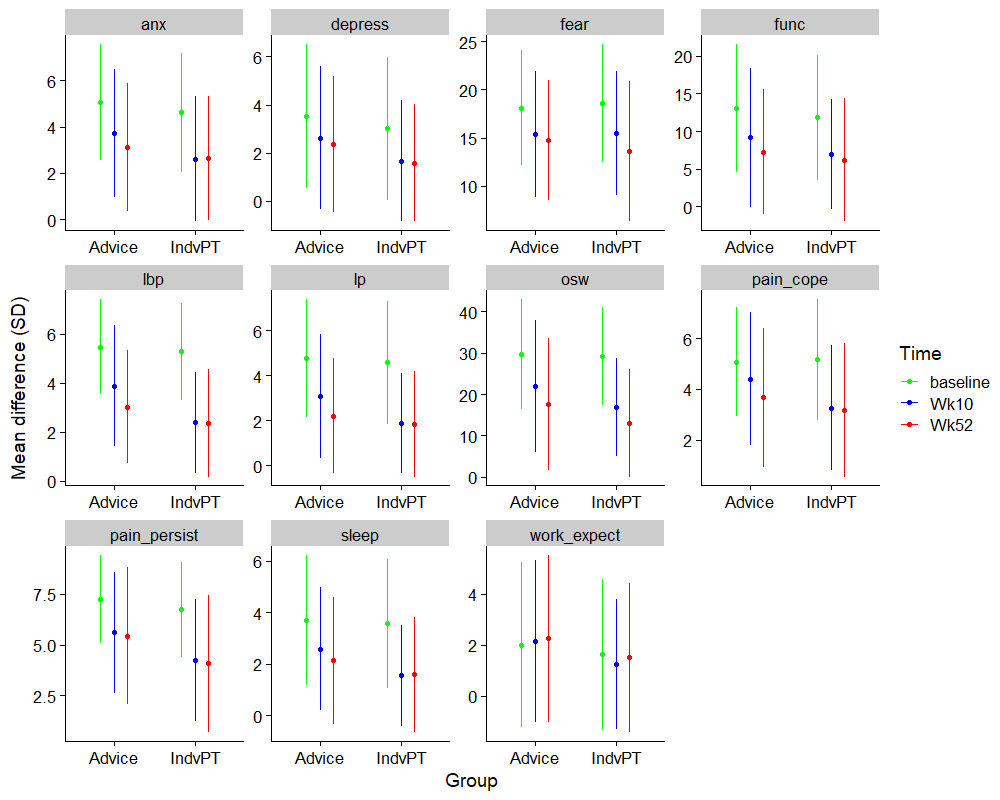

Supplement: S2 Fig — Abbreviation: Anx—anxiety; lbp–low back pain; lp–leg pain; IndvPT–individualized physiotherapy. (TIFF) [file pone.0258515.s002.tiff]
